# The impact of lesion side on bilateral upper limb coordination after stroke

**DOI:** 10.1186/s12984-023-01288-4

**Published:** 2023-12-13

**Authors:** Pei-Cheng Shih, Christopher J. Steele, Dennis Hoepfel, Toni Muffel, Arno Villringer, Bernhard Sehm

**Affiliations:** 1 Department of Neurology, Max Planck Institute for Human Cognitive and Brain Sciences, Leipzig, Germany; 2 Sony Computer Science Laboratories, Inc, Tokyo, Japan; 3 Department of Psychology, Concordia University, Montreal, QC Canada; 4 Clinic and Polyclinic for Psychiatry and Psychotherapy, Leipzig, Germany; 5 Charité Universitätsmedizin Berlin, Berlin, Germany; 6 Department of Cognitive Neurology, University Hospital Leipzig, Leipzig, Germany; 7 Department of Neurology, University Hospital Halle (Saale), Halle, Germany

**Keywords:** Upper extremity, Rehabilitation, Stroke, Movement kinematic, Synchronization

## Abstract

**Background:**

A stroke frequently results in impaired performance of activities of daily life. Many of these are highly dependent on effective coordination between the two arms. In the context of bimanual movements, cyclic rhythmical bilateral arm coordination patterns can be classified into two fundamental modes: in-phase (bilateral homologous muscles contract simultaneously) and anti-phase (bilateral muscles contract alternately) movements. We aimed to investigate how patients with left (LHS) and right (RHS) hemispheric stroke are differentially affected in both individual-limb control and inter-limb coordination during bilateral movements.

**Methods:**

We used kinematic measurements to assess bilateral coordination abilities of 18 chronic hemiparetic stroke patients (9 LHS; 9 RHS) and 18 age- and sex-matched controls. Using KINARM upper-limb exoskeleton system, we examined individual-limb control by quantifying trajectory variability in each hand and inter-limb coordination by computing the phase synchronization between hands during anti- and in-phase movements.

**Results:**

RHS patients exhibited greater impairment in individual- and inter-limb control during anti-phase movements, whilst LHS patients showed greater impairment in individual-limb control during in-phase movements alone. However, LHS patients further showed a swap in hand dominance during in-phase movements.

**Conclusions:**

The current study used individual-limb and inter-limb kinematic profiles and showed that bilateral movements are differently impaired in patients with left vs. right hemispheric strokes. Our results demonstrate that both fundamental bilateral coordination modes are differently controlled in both hemispheres using a lesion model approach. From a clinical perspective, we suggest that lesion side should be taken into account for more individually targeted bilateral coordination training strategies.

*Trial registration*: the current experiment is not a health care intervention study.

**Supplementary Information:**

The online version contains supplementary material available at 10.1186/s12984-023-01288-4.

## Background

Although various effective rehabilitation programs have been developed over the past decades, over half of chronic stroke patients still experience difficulty in achieving daily activities with their upper limbs [1]. Upper limb impairments can be characterized by impaired control of movement of the contralesional arm and difficulty in coordinating the limbs, both of which impact on quality of life [2, 3]. Rehabilitation after stroke focuses predominantly on treating the contralesional arm. However, our daily activities are highly dependent on the coordination between the two arms, and this has received far less attention [4]. Understanding the characteristics and mechanisms of bilateral coordination impairments after hemiparesis is therefore crucially needed to develop effective rehabilitation strategies.

Among the broad repertoire of human upper-limb bilateral coordination patterns, cyclic rhythmical bilateral movements can be classified into two fundamental modes: in-phase (i.e., bilateral homologous muscles contract simultaneously) and anti-phase (i.e., homologous muscles contract alternately) movements [5]. Research in healthy young adults has established that anti-phase movements are more complex and unstable [6, 7]. During anti-phase movements, participants display higher spatial and temporal variability and worse inter-limb synchronization compared to in-phase movements [8–10]. Though both in-phase and anti-phase movements require bilateral coordination, research in healthy adults suggests that they are associated with different control mechanisms. During bilateral in-phase finger movements, previous research has found increased activation of the left (dominant) hemisphere compared to the right [11, 12], as well as causal information flow of the BOLD signal from the left to the right motor cortex [13]. These suggest the dominant role of the left hemisphere in bilateral in-phase movement execution. In contrast, no significant laterality effects were observed during bilateral anti-phase finger movements, which suggest a similar contribution from the two hemispheres [14, 15].

After stroke affecting the motor system, patients generally exhibit greater movement variability [16] and unsteady force control [17] during bilateral movements, regardless of the coordination patterns. Also, the interhemispheric balance between the two hemispheres as well as the laterality in ascending pathways showed changes during bilateral movements after stroke [18, 19], indicating neural reorganization in bilateral movement execution [20, 21]. Moreover, like healthy adults, stroke patients experience more difficulty performing anti-phase than in-phase movements [22, 23]. However, considering that the two hemispheres are differentially involved in in- and anti-phase movements in healthy adults, we would expect distinct characteristics in bilateral coordination impairments after left and right hemispheric stroke. Consistent with this, one previous study found that left hemispheric stroke (LHS) compared to right hemispheric stroke (RHS) patients showed better inter-limb synchronization during in-phase elbow pronation-supination movements [24]. However, given that bilateral coordination is controlled by a complex system comprising both individual-limb and inter-limb components [25], a successful bilateral movement requires not only a good inter-limb coordination but also an accurate individual-limb control. Therefore, it is still unclear from the literature how individual limb performance during bilateral movements is affected after left and right hemispheric stroke.

To examine whether the lesion hemisphere influences bilateral movements after stroke, we compared inter- and individual-limb performance between stroke survivors with left and right hemispheric lesions. Inter-limb performance was examined using inter-limb synchronization index, and individual-limb by movement trajectory variability of the contralesional and ipsilesional hands. We expected that patients in general exhibit deficits in bilateral movement performance. However, we hypothesized to find differences between both groups (left vs. right hemispheric lesions). Specifically, we hypothesized firstly, that left hemispheric stroke patients exhibit stronger impairments in bilateral in-phase coordination compared to right hemispheric stroke patients. This is based on the observed left hemispheric dominance during bilateral in-phase movements in the healthy population [11]. Secondly, since right hemispheric stroke leads to a larger imbalance in interhemispheric inhibition (IHI) compared to left hemispheric stroke [26], we hypothesized that bilateral anti-phase movements, which require balanced activity in bilateral hemispheres, would exhibit stronger impairments in patients with right hemispheric stroke. Also, we furthermore assessed the individual contributions of the two hands to inter-limb synchronization. Specifically, we examined whether it is possible to use the individual-limb performance from both arms to predict the inter-limb coordination control in patients with left- and right-hemispheric stroke. This enabled us to use hand performance to infer how motor stroke affects the differential roles of the two hemispheres in bilateral coordination.

## Methods

### Participants

The ethics committee of the University of Leipzig approved the study protocol, and participants were given informed consent before their eligibility assessment. Patients were recruited from two sources: the Day Clinic for Cognitive Neurology in University Hospital Leipzig, and advertisements on the local newspaper Leipziger-Volkszeitung. A total of 60 stroke patients were invited and screened between September 2016 and September 2018 for eligibility. The inclusion criteria were: (1) first onset of stroke resulting in hemiparesis; (2) chronic phase after stroke (> 6 months from stroke incident); (3) mild-to-moderate motor impairment (Fugl-Meyer-Upper Limb Score; FM-UE > 20 points); (4) No elbow spasticity (Modified Ashworth Scale, MAS < 3). (5) Able to understand and follow the instruction correctly inside the KINARM. The exclusion criteria were: (1) any other kind of systematic and neurological diseases; (2) cognitive deficits (Mini-Mental Scale Examination; MMSE < 24); (3) contraindication for MRI; (4) unilateral neglect or other visual impairment.

Eighteen (nine left and nine right hemispheric stroke) patients fitted the criteria and were included in the experiment. All patients were right-handers before the stroke (note: handedness before stroke was not a criteria for this experiment). After the patients’ recruitment, 18 age- and sex-matched healthy-control adults were identified from the database of the Max Planck Institute for Human Cognitive and Brain Sciences, and participated in the study. The inclusion criteria for the control group were: (1) right-handedness; (2) no known diseases. Participants were classified into four subgroups (see Table 1 for demographics): left hemispheric stroke (LHS), right hemispheric stroke (RHS), the control group for the left hemispheric stroke (LHC), and the control group for the right hemispheric stroke (RHC).

**Table 1:** Demographic data of the participants

Variables/groups	Stroke patients		Healthy controls	
Group label	LHS (n = 9)	RHS (n = 9)	LHC (n = 9)	RHC (n = 9)
Lesioned hemisphere	Left	Right	NA	NA
Age	54.7 ± 14.4	60.8 ± 13.3	54.7 ± 14.4	60.8 ± 13.3
Sex (M/F)	5/4	3/6	5/4	3/6
Year since stroke	9.0 ± 5.1	5.4 ± 3.2	NA	NA
Stroke type (I/H)	8/1	8/1	NA	NA
Lesion (% of brain volume)	5.5 ± 7.5	9.5 ± 12.4	NA	NA
MMSE	28.9 ± 1.9	29.9 ± 0.3	30.0 ± 0.0	29.9 ± 0.3
FM-UE	48.9 ± 11.8	41.8 ± 13.9	NA	NA
NIHSS	2.6 ± 2.3	2.8 ± 1.4	NA	NA
MAS (0/1/1 + /2)	1/5/2/1	0/6/1/2	NA	NA
Proprioception error (cm)	5.1 ± 4.1	5.6 ± 2.4	NA	NA

Data is presented as mean ± SD of each group

*LHS* left hemispheric stroke, *RHS* right hemispheric stroke, *LHC* left hemispheric control, *RHC* right hemispheric control, *I* ischemic stroke, *H* hemorrhagic stroke, *MMSE* Mini-Mental Scale Examination, *FM-UE* Fugl-Meyer-Upper Limb Score, *NIHSS* National Institutes of Health Stroke Scale, *MAS* Modified Ashworth Scale

### Clinical examination for stroke patients

For the stroke patients, clinical tests for quantifying motor, sensory, and cognitive impairments were documented during the screening day by a board-certified neurologist (BS) and a physical therapist (PCS). The screening examination involved a thorough clinical neurological assessment including Visual Field Testing to rule out deficits that could confound the results such as hemineglect. Furthermore, clinical observations (such as testing patients’ spontaneous orientation), Wiggle test [27], and clock drawing test [28] were used to identify visual extinction and visual neglect. Besides the tests used as inclusion/exclusion criteria (i.e., MMSE, MAS, FM-UE, as described in the “Participants” section), NIHSS (National Institutes of Health Stroke Scale) and proprioception of the elbow flexors were also documented. Proprioception ability was quantified by the Arm Position Matching test (please see Additional file 1) using the KINARM upper limb robotic exoskeleton system (BKIN Technologies, Canada).

### Device and task

The experiment was conducted using the KINARM system. Participants sat within the KINARM on an adjustable chair and faced an augmented-reality screen. Both arms rested on gravity-support platforms, and both hands held cylinder grips. For stroke patients, an extra elastic band was wrapped around the hand and the grip to provide more support at the endpoint.

**Fig. 1:**
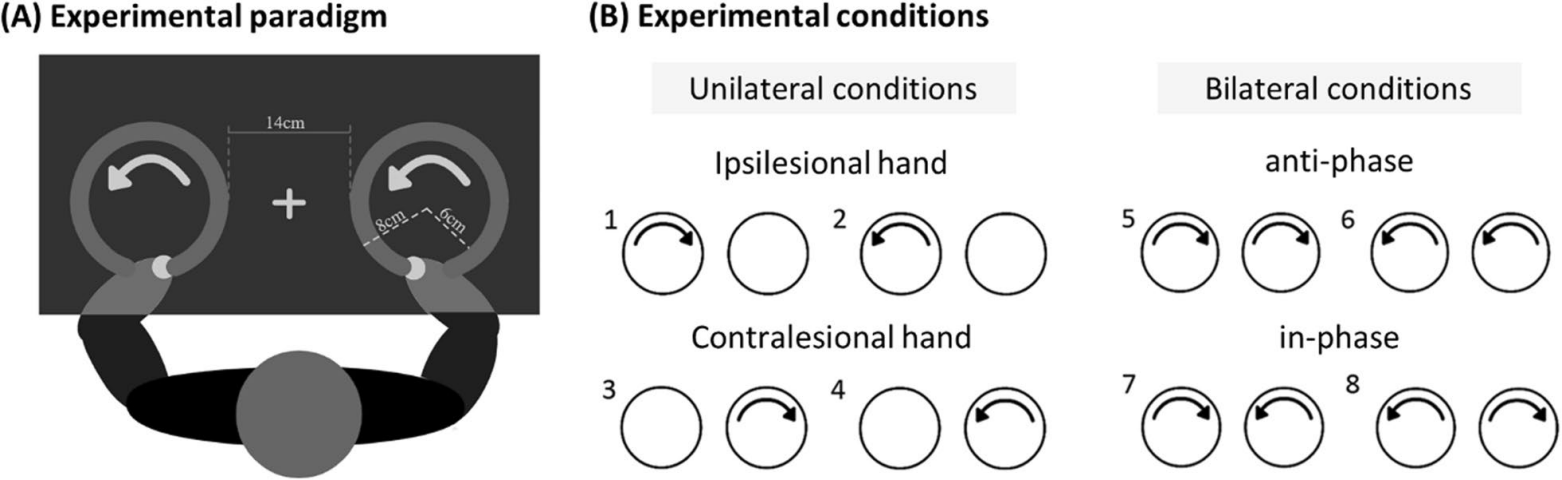
Experimental design. **A** Experimental setup. The augmented-reality screen displayed the paradigm. The white fixation cross was presented at the midline of participants. **B** Experimental conditions. There were four main conditions in the experiment, each with movements to the left and right. 1–2: unilateral movements with the ipsilesional arm, 3–4: unilateral movements with the contralesional arm, 5–6: bilateral anti-phase movements, 7–8: bilateral in-phase movements

We adapted the classic bilateral circle drawing task [29] that was also used in our previous study [30]. Two side-by-side circles, indication arrow(s) inside the circle(s), and a fixation cross were displayed on the screen (Fig. 1A). A bar was placed at the top of each circle to indicate the starting position. There were eight movement conditions in the experiment, classified into four movement patterns (Fig. 1B): unilateral movement of the ipsilesional hand, unilateral movement of the contralesional hand, bilateral anti-phase movements, and bilateral in-phase movements. Each trial consisted of a preparation phase and a movement phase. In the preparation phase, participants put both hands on the starting points and confirmed the upcoming condition. After 5 s, an auditory metronome indicated the start of a 15-s movement phase. During the movement phase, participants were instructed to draw continuous circles in synchrony with the metronome. The frequency of the metronome was adjusted to the capabilities of the groups. A separate pilot experiment determined the maximum movement speed for each group without phase transitions. Phase transition refers to the observed phenomenon that anti-phase movements could unintentionally change to in-phase movements as movement frequency is increased [31, 32]. Based on the pilot results, 0.85 Hz was used for healthy controls (12 complete movement cycles per block) and 0.75 Hz for stroke patients (11 complete movement cycles per block). These movement frequencies allowed both groups to generate consistent cyclic rhythmic movements within each trial throughout the experiment. During the experiment, participants were instructed to focus their eyes on the fixation cross at the center during movements to reduce potential attentional bias toward a specific side [8].

Each condition was performed once in a randomized order during each block, and there were ten blocks in each experiment. To reduce the occurrence of fatigue during the experiment, a 2-min break was set between blocks 5 and 6. Before the experiment started, all participants practiced every movement condition once for familiarization with the task.

### Kinematic data recording and processing

Participants’ hand movements were continuously recorded at a sampling rate of 1000 Hz with KINARM using Dexterit-E (v3.5, BKIN Technologies, Canada). The recorded data included both x and y coordinates of the hand position. Data were imported into Matlab R2017b (The MathWorks, USA) for further processing.

To examine task performance during bilateral movements (condition 5–8), we computed two indices for each trial to represent individual-limb and inter-limb performance, respectively: (1) trajectory variability and (2) inter-limb synchronization [10]. Conditions under each category were considered as the same condition in the statistical analysis; for example, condition 5 and 6 are marked as anti-phase condition, and conditions 7 and 8 are marked as in-phase condition. For unilateral conditions (condition 1–4), only trajectory variability was computed.

#### Trajectory variability (individual-limb)

Movement trajectories were converted from Cartesian (*x*, *y*) to polar coordinates, and the radius (*r, distance from the center of circle*) was extracted from each sample. Trajectory variability was calculated as the coefficient of variation of all radii values within each trial and for each hand separately. A lower value represents a more consistent movement trajectory during the task.

#### Inter-limb synchronization (Inter-limb)

Inter-limb synchronization represents how well the two hands were synchronized with each other during bilateral movements. We extracted the phase value (*θ*) of each sample from both hands, and used the classic phase synchronization index [33] to quantify the inter-limb synchronization:

1 $$Inter{\text{-}}limbsynchronization=\left|\frac{1}{T}{\sum }_{t=1}^{T}{e}^{i\left[{\varphi }_{R}\left(t\right)-{\varphi }_{L}\left(t\right)\right]}\right|,$$

where ${\varphi }_{R}\left(t\right)$ and ${\varphi }_{L}\left(t\right)$ represent the unwrapped phase of the right and left hand during each sample $t$, and $T$ represents the total number of sampling points in a given trial. This index ranges from 0 to 1, with 1 corresponding to perfect phase synchronization.

### Lesion assessment

Structural imaging data were acquired on a 3T Skyra-MR scanner (Siemens, Germany). The scanning sequences included MP2RAGE (FoV = 256 × 256 mm, TR = 5000 ms, TE = 2.9 ms, TI1 = 700 ms, TI2 = 2500 ms, flip angle 1 = 4°, flip angle 2 = 5°, slice thickness = 1 mm) and FLAIR (FoV = 220 × 220 mm, TR = 10000 ms, TE = 93 ms, TI = 2500 ms, flip angle = 180°, slice thickness = 4 mm). Lesions were semi-automatically mapped from the FLAIR images using Clusterize Toolbox [34], and the lesion volume of each participant was calculated with the same toolbox. All lesion maps were normalized to the MNI space to compute the lesion conjunction map (Additional file 1).

### Statistical analysis

#### Demographic data

Demographic data including age, time since stroke, lesion volume, FM-UE, MMSE, NIHSS, and proprioception error between the two stroke groups (RHS and LHS) were compared using two sample t-tests. Ordinal data, i.e. MAS, was compared using ordinal logistic regression.

#### Task performance: trajectory variability (individual-limb)

Kinematic data were analyzed with linear mixed-effects models (LMM), which allow better control for random sources of variance without the loss of statistical power resulting from data aggregation across subjects [35]. As stroke samples often have high inter-subject variability, LMM offers a better approach than univariate ANOVA or ordinary least squares regression for modeling heteroscedasticity and minimizing the outlier effects from individual subjects.

All mixed-effects analyses were conducted with Rstudio (v3.0.2) using the *lme4* (v1.1-18.1) package [36] for modeling and the *emmeans* (v1.4.1) package for pairwise comparison between factors. We inspected residuals of each linear mixed model using QQ-plots. Despite slight deviations from the normal distribution, we nevertheless chose to report our model results, since linear mixed models have been shown to be robust against non-normality [37].

To examine whether left- and right-hemispheric stroke affected motor performance differently, we considered *Group* (Stroke/Control) and *Lesioned Hemisphere* (LH/RH) as between-subject fixed effects, with a random intercept for each subject for the unilateral conditions. For the bilateral conditions, we additionally included Condition (anti-phase or in-phase) as a within-subject fixed effect. For both unilateral and bilateral movements, pairwise comparisons were performed between *Group* in each *Lesioned Hemisphere* with Bonferroni correction for multiple comparisons. Contralesional and ipsilesional hand performances were analyzed separately. Importantly, since the performance of the right hand is generally better than the left hand in right-handers, the hands of the two control groups were matched to their respective stroke patient groups. For example, the contralesional hand (i.e. right hand) performance of left-hemispheric stroke patients was compared to the right hand of their matched control group, while the contralesional hand (i.e. left hand) performance of right-hemispheric stroke patients was compared to the left hand of their matched control group.

#### Task performance: inter-limb synchronization (inter-limb)

Akin to analyzing trajectory variability in the bilateral conditions, the model for inter-limb synchronization consisted of three fixed factors *Group* (stroke/ control), *Lesioned Hemisphere* (left/right)*,* and *Condition* (anti-/in-phase), with random intercepts for each participant. Pairwise comparisons were performed between *Group* in each *Lesioned Hemisphere,* Bonferroni corrected for multiple comparisons.

#### Effects of individual-limb performance on inter-limb synchronization

To characterize how the contribution of both hands change in bilateral coordination after left and right hemispheric stroke, we examined the effect of individual-limb (trajectory variability) on inter-limb (inter-limb synchronization) parameters.

For both bilateral in-phase and anti-phase movements, we first performed regression analyses in the pooled healthy control participants to determine the normative contributions of dominant and non-dominant hands to different bilateral coordination patterns. We built a linear mixed regression model to test the role of hand-dominance in the relationship between individual limb control and inter-limb synchronization separately for in-phase and anti-phase movements. This is modelled by the formula Inter-limb synchronization ~ Trajectory variability*Hand (dominant/non-dominant hand) + (1|Subject), which includes the interaction between Trajectory variability and Hand and lower order terms as fixed effects, and a random intercept for each subject. After establishing the normative relationship, we then examined stroke patients and compared whether this prediction differs between the two stroke groups using the model: *Inter-limb synchronization* ~ Trajectory variability*Hand (contralesional/ipsilesional hand)*Lesioned hemisphere (LHS/RHS) + (1|*Subject*). In the case of interaction effects, data were visualized using *jtools* (v2.0.3), and pairwise comparisons were performed between *Hand* in each *Lesioned Hemisphere* with Bonferroni correction for multiple comparisons.

## Results

### Demographic data

The levels of impairment in our stroke patients ranged from mild to moderate severity (Table 1). There were no statistically significant differences in age (t = − 0.94, p = 0.36), duration of time since stroke (t = − 1.82, p = 0.09), lesion volumes (t = − 0.83, p = 0.42), FM-UE (t = 1.18, p = 0.25), MMSE (t = − 1.56, p = 0.16), NIHSS (t = − 0.27, p = 0.80), proprioception ability (t = − 0.08, p = 0.94), and MAS (LR = 0.39, p = 0.53) between the two stroke groups.

### Task performance: trajectory variability (individual-limb control)

All data of trajectory variability are summarized in Table 2.

**Table 2:** Average trajectory variability in each group and condition

Variables	Left hemispheric lesion		Right hemispheric lesion	
Groups	Stroke (LHS)	Control (LHC)	Stroke (RHS)	Control (RHC)
Contralesional hand	Right hand	Right hand	Left hand	Left hand
Unilateral conditions	0.19 ± 0.42*	0.12 ± 0.03	0.19 ± 0.04*	0.15 ± 0.03
Bilateral anti-phase	0.21 ± 0.05	0.13 ± 0.03	0.28 ± 0.08*	0.18 ± 0.05
Bilateral in-phase	0.21 ± 0.07*	0.12 ± 0.01	0.23 ± 0.07	0.16 ± 0.03
Ipsilesional hand	Left hand	Left hand	Right hand	Right hand
Unilateral conditions	0.16 ± 0.03	0.13 ± 0.03	0.14 ± 0.03	0.12 ± 0.02
Bilateral anti-phase	0.19 ± 0.03	0.17 ± 0.06	0.18 ± 0.04	0.14 ± 0.02
Bilateral in-phase	0.17 ± 0.03	0.15 ± 0.03	0.16 ± 0.04	0.13 ± 0.02

Data is presented as mean ± SD of each group and condition

*LHS* left hemispheric stroke, *RHS* right hemispheric stroke, *LHC* left hemispheric control, *RHC* right hemispheric control

Please note that, due to handedness, performance of the right hand is generally better than the left hand in the control groups. *p < 0.05 compared to control group (after controlled for multiple comparisons)

#### Unilateral movements

**Fig. 2:**
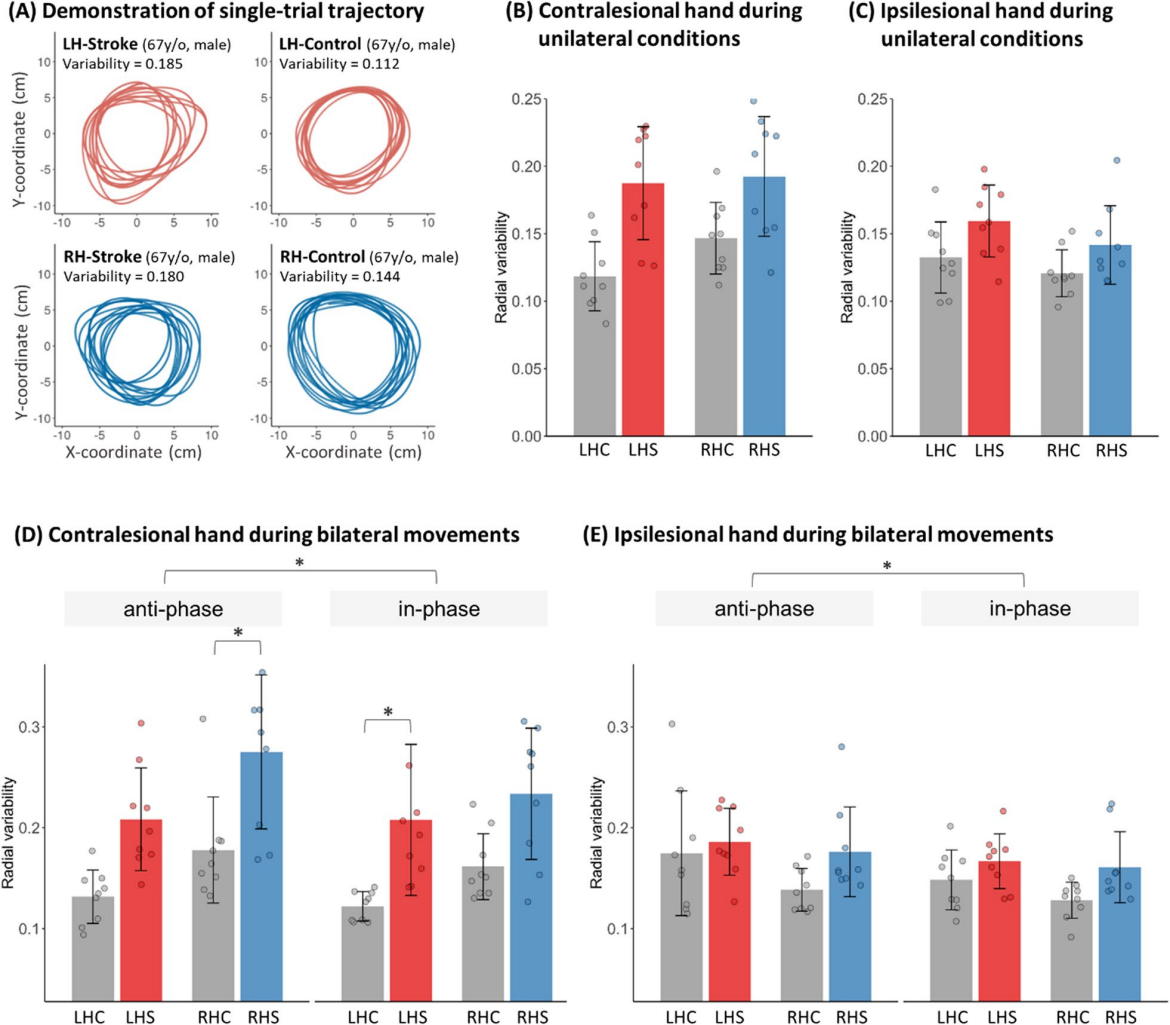
Trajectory variability in the two stroke and two control groups. **A** An example trajectory plot of the contralesional arm from one representative participant of each group. **B** Trajectory variability of the contralesional hand during unilateral conditions on the group level. Both LHS and RHS patients showed higher trajectory variability compared to their control groups. **C** Trajectory variability of the ipsilesional hand during unilateral conditions. Stroke patients showed higher trajectory variability compared to their control groups, but no pairwise comparisons survived the correction of multiple comparisons. Translucent points: individual mean data. **D** Performance of the contralesional hand. Generally, patients showed higher trajectory variability compared to the control groups. Specifically, RHS patients displayed stronger impairment during anti-phase movements, while LHS patients had more impairments during in-phase movements. **E** Performance of the ipsilesional hand. Stroke patients showed higher trajectory variability compare to the control groups, but no significant interaction with the lesion side. No pairwise comparisons survived the statistical threshold after corrected for multiple comparisons. Translucent points: individual mean data. *LHC* left hemispheric control, *LHS* left hemispheric stroke, *RHC* right hemispheric control, *RHS *right hemispheric stroke. *p < 0.05 compared to the control group (after corrected for multiple comparisons). Column and error bar = mean ± se

We found no significant differences in unilateral movement performance in patients with left and right hemispheric lesions (Fig. 2A, B), indicating that their unilateral movement impairment levels were similar (please refer to Additional file 1: Material 3 for a more detailed description).

#### Bilateral movements

Figure 2D depicts the trajectory variability of the contralesional hand during bilateral in-phase and anti-phase movements. Generally, regardless of stroke or not, participants showed higher trajectory variability during anti-phase compared to in-phase conditions. Also, stroke patients showed higher trajectory variability compared to controls. However, specifically, LHS patients showed more impairments during in-phase movements while RHS patients showed more impairments during the anti-phase movements. This was revealed by the mixed 2 × 2 × 2 LMM, with a significant Group*Lesioned Hemisphere*Condition interaction (F = 16.71, p < 0.001), and main effects of *Group* (F = 23.28, p < 0.001), *Lesioned Hemisphere* (F = 6.80, p = 0.01) and *Condition* (F = 72.00, p < 0.001). Pairwise comparisons showed significant differences between LHC and LHS in the in-phase (t = − 3.49, p = 0.027) but non-significant (though borderline) differences in the anti-phase (t = − 3.15, p = 0.060) condition. In contrast, RHS patients were significantly impaired in the anti-phase (t = − 3.95, p = 0.008) but not the in-phase (t = − 2.96, p = 0.09) condition, relative to RHC. Figure 2E shows the trajectory variability of the ipsilesional hand. The LMM revealed significant main effects of *Group* (F = 5.35 p = 0.027) and *Condition* (F = 84.07, p < 0.001), but not *Lesioned Hemisphere* (F = 0.105, p = 0.11), without a three-way interaction (F = 1.96, p = 0.162). These results indicated that anti-phase, compared to in-phase movements, had higher variability regardless of group.

Taken together, when examining the contralesional hand, anti-phase movements were found to be more affected in the RHS group, while in-phase movements were more affected in the LHS group compared to their control groups, respectively. As for the ipsilesional hand, no lesioned-hemispheric-dependent effects were found.

### Task performance: inter-limb synchronization (inter-limb)

**Fig. 3:**
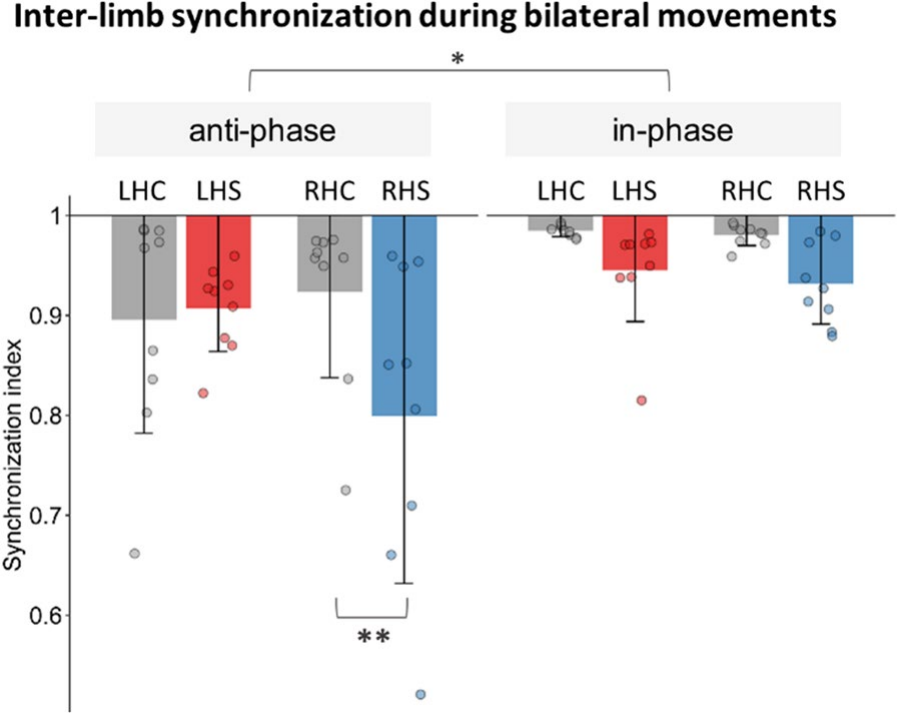
Inter-limb synchronization performance during bilateral movements, quantified using the synchronization index. Inter-limb synchronization value ranges from 0 to 1, with 1 indicating perfect synchronization (good performance) and 0 indicating no synchronization (poor performance). A Group*Lesioned Hemisphere*Condition interaction was found. Pairwise comparisons revealed that RHS patients displayed more impairments during anti-phase movements. Translucent points: individual mean data. *LHC* left hemispheric control, *LHS* left hemispheric stroke, *RHC* right hemispheric control, *RHS* right hemispheric stroke. **p < 0.01. Column and error bar = mean ± se

Figure 3 shows the inter-limb synchronization during bilateral movements. Similar to the trajectory variability, stroke patients generally showed worse performance compared to controls. Moreover, the RHS group had additional impairments during anti-phase movements, revealing by a significant Group*Lesioned Hemisphere*Condition interaction (F = 54.47, p < 0.001). Main effects in *Group* (F = 5.50, p = 0.025) and *Condition* (F = 390.23, p < 0.001), but not *Lesioned Hemisphere* (F = 1.26, p = 0.27) were also observed. Pairwise comparisons showed no significant differences between LHC and LHS in both anti-phase (t = − 0.34, p = 1.000) and in-phase (t = 1.29, p = 0.895) movements. In contrast, there was a significant difference in the anti-phase (t = 3.88, p = 0.01), but not in-phase (t = 1.69, p = 0.69) condition in RHS relative to RHC group. These results show that regardless of group, both hands were more synchronized during the in-phase compared to anti-phase condition. Also, the stroke groups showed less inter-limb synchronization compared to controls. Most importantly, patients with right hemispheric lesion specifically showed impairment in coordinating the two hands during anti-phase movements.

### Effects of individual-limb performance on inter-limb synchronization

#### Anti-phase conditions

Regression analyses revealed that the performance of the two hands showed similar strength in predicting inter-limb behavior during anti-phase movements for the control participants and the two stroke groups. Please refer to Additional file 1: Materials 4 for a more detailed description.

#### In-phase conditions

Regression analyses revealed that in both healthy controls and RHS patients, the performance of right hand, compared to left, is a stronger predictor in predicting inter-limb behavior, whilst vice versa in LHS patients.

**Fig. 4:**
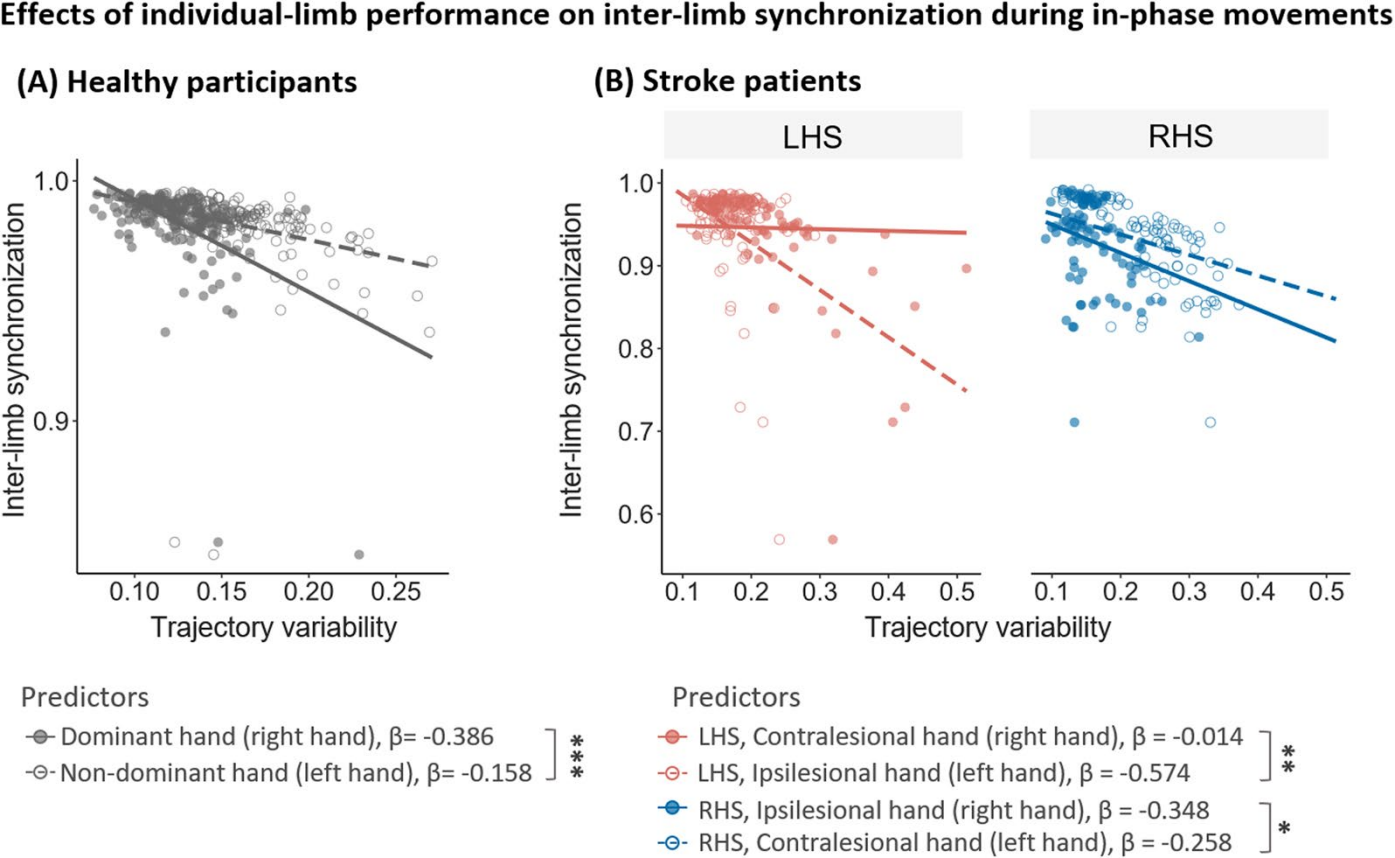
Effects of individual-limb performance on inter-limb synchronization during in-phase movements. Each dot represents the performance of each trial in each participant. **A** In healthy controls, the dominant hand was more predictive of inter-limb performance compared to the non-dominant hand. **B** In stroke patients, the paretic hand was a stronger predictor of the change inter-limb

For healthy controls (Fig. 4A), the regression model revealed a significant effect of *Hand* (df = 352.28, F = 13.76, p < 0.001) such that the dominant hand (β_dominant_ = − 0.386) was significantly better at predicting inter-limb synchronization compared to the non-dominant hand (β_non-dominant_ = − 0.158).

For stroke patients (Fig. 4B), there was an interaction between *Hand* and *Lesioned Hemisphere* (df = 342.13, F = 5.85, p = 0.016), a main effect of *Hand* (df = 342.13, F = 11.45, p < 0.001), but no main effect of *Lesioned Hemisphere* (df = 348.95, F = 0.0001, p = 0.99). Pairwise comparisons revealed that in both stroke groups, contralesional hand performance was a significantly better predictor of the inter-limb synchronization, and that the interaction was driven by stronger prediction in LHS patients (β_LHS,contralesional_ = − 0.574, β_LHS,ipsilesional_ = − 0.014, t = − 2.64, p = 0.009) than RHS (β_RHS,contralesional_ = − 0.348, β_RHS,ipsilesional_ = − 0.258, t = − 2.24, p = 0.03). Notably for both the healthy controls and the RHS groups, the right hand (the dominant/ipsilesional hand) was the strongest predictor of inter-limb synchronization. In contrast, for the LHS group, the left hand (ipsilesional) predicted inter-limb performance more strongly.

Taken together, performance of both hands showed similar strength in predicting inter-limb synchronization performance during anti-phase movements in both stroke patients and controls. However, during in-phase movements right hand (dominant hand) performance predicted inter-limb synchronization behavior more strongly than the left hand (non-dominant) in healthy controls. This same effect was found in patients with right-hemispheric lesions, indicating that this mechanism is preserved. However, in stroke patients with left-hemispheric lesions, the prediction was reversed such that left hand (ipsilesional) performance was a stronger predictor of inter-limb synchronization during in-phase movements. These results indicate a shift in hand contribution in the LHS group.

## Discussion

The present study sought to use kinematic measures to determine how lesion hemisphere affects individual-limb and inter-limb controls during bilateral movements in stroke survivors. We examined stroke patients with left and right hemispheric lesions as they performed a bilateral circle drawing task, and observed lesioned hemisphere-dependent impairments. Patients with right hemispheric lesions showed more impairment in controlling anti-phase movements, while patients with left lesions were more affected in in-phase movements. These results suggest that the different roles of the two hemispheres during in-phase and anti-phase movements lead to differentially reduced bilateral coordination performance after left and right hemispheric stroke.

More specifically, first, for the trajectory variability during bilateral movements, the RHS group showed pronounced deficits in anti-phase movements, while the LHS group showed slightly more impairment in in-phase movements. Second, in the inter-limb assessment (inter-limb synchronization), the RHS group notably displayed worse coordination ability during anti-phase compared to the LHS group, but did not show significant differences in the in-phase condition: both stroke groups showed only mild impairments in hand-coordination during in-phase movements. These results suggest differential impairments after left and right hemispheric stroke in bilateral coordination. However, previous studies have shown that impairments in bilateral movements could be driven by specific impairments in motor control [38–40] or asymmetrical proprioceptive ability of the affected limb [41, 42]. To rule out these potential contributions, we examined the impairment levels of affected-side movements (Additional file 1: Materials 3) and proprioceptive ability (Additional file 1: Materials 1) and found no significant differences between the two patient groups. Therefore, we are confident that the results from our current experimental setup are specific to bilateral movements.

### Left hemispheric stroke leads to impairments in bilateral in-phase movements

Why are in-phase movements more affected in the LHS group? Early studies have suggested that the left (dominant) hemisphere plays a major role in organizing coupled bilateral finger movements, and less so in the right (non-dominant) hemisphere [11, 43]. During in-phase finger movements, the left hemisphere showed larger task-related BOLD signal changes compared to the right hemisphere, while during anti-phase movements, the two hemispheres showed similar BOLD changes [15]. Furthermore, transcranial magnetic stimulation (TMS) pulses applied to the left hemisphere have been shown to interrupt movements of both hands during in-phase movements, but not anti-phase movements [44]. These studies therefore provide evidence that in-phase movements are organized in the left hemisphere. Consistent with this, we found significant impairments in controlling the trajectory of the contralesional hand during in-phase movements in the LHS group. However, when looking at the inter-limb synchronization during in-phase movements, LHS and RHS patients showed similar levels of impairment. One possibility is that the individual-limb measurement is a spatial measure, while the inter-limb measurement is a temporal measure. Another explanation could be that while the trajectory variability only considered individual limb performance, the synchronization index involved both limbs. Therefore, to resolve the discrepancy between inter- and individual-limb performances in LHS patients, we further examined how the two hands contributed to inter-limb coordination.

By examining how individual-limb performance predicts inter-limb performance, we first detected in the healthy controls that the right hand contributed more during the in-phase movements compared to the left hand (Fig. 4A). This implies a predominant contribution of the dominant side to the in-phase movements in healthy participants, which is in line with the findings from previous neuroimaging studies [13, 15, 44].

In stroke patients (Fig. 4B), we found that the relative contribution of the hands to the inter-limb coordination depends on the side of the lesioned hemisphere. RHS patients displayed a similar intra/inter-limb relationship as healthy participants: right hand (ipsilesional) performance predicted inter-limb synchronization better than left hand (contralesional) performance. This supports the view that in-phase movements are driven by left hemisphere centralized control. In contrast, for patients in the LHS group we found that left hand (ipsilesional) performance was a stronger predictor of inter-limb synchronization compared to the right (contralesional) hand. This result is particularly important, as it shows that patients with left hemispheric lesions might change their main contributing hemisphere from the left (lesioned) to the right during bilateral in-phase movements.

Two explanations may be considered, why the main contributing hand shifted from the right to the left during in-phase movements in the LHS group (Fig. 4). One possibility (i) could be the switching of preferred hand during daily living from the left to the right hand after a left hemispheric stroke [45]. However, this is unlikely as we would have observed the shift in hand contribution in both in-phase and anti-phase movements. A more likely alternative (ii) is that this reversal in hand contribution may be a result of neural compensation to the damaged left hemisphere. Since the left hemisphere, which plays a leading role for in-phase movements, was impaired after stroke, the right hemisphere takes over the responsibility for guiding this movement. This compensatory shift could explain why an increased impairment in inter-limb synchronization was not observed in the LHS group during in-phase movements. Further neuroimaging studies will need to confirm this theory.

### Right hemisphere stroke leads to impairments in bilateral anti-phase movements

Contrary to in-phase movements, anti-phase movements require a more balanced relationship between the two hemispheres. Since the bilateral homologous muscles are not activated simultaneously, contralateral movement suppression is needed [46]. Likewise, successful anti-phase movement performance is characterized by bidirectional information flow between the hemispheres in healthy participants [13]. Consistent with this, we demonstrated that for all participants, regardless of group, the two hands similarly predicted the inter-limb synchronization during anti-phase movements. This suggests an equal contribution of the two hemispheres to inter-limb coordination behavior during anti-phase movements, regardless of the stroke side.

We further demonstrated that patients with RHS exhibit worse individual-limb trajectory accuracy and inter-limb synchronization during anti-phase movements than those with LHS—but what is the plausible mechanism behind it? Previous studies have shown that functional interactions between the hemispheres are more imbalanced in patients with right hemispheric stroke than left hemispheric stroke [26]; that is, for patients who were right-hand dominant before stroke, greater interhemispheric inhibition (IHI) is directed from the left to right hemisphere compared to the other way around. This means the suppression of contralesional hemisphere activation is more challenging in RHS compared to LHS group [47]. In healthy right-handers, the right motor cortex originally has a lower capacity to inhibit the left motor cortex than vice versa [48, 49], and this effect becomes larger in RHS patients compared to LHS [47], which results in a more imbalanced interhemispheric relationship. However, asymmetries in interhemispheric transfer are also less influential during voluntary muscle activation compared to when muscles are at rest [26]. Therefore, how the individual interhemispheric inhibition changed during anti-phase movements in the RHS patients should be examined in future studies to address the importance of a balanced inter-hemisphere relationship in efficient anti-phase movements.

Greater impairment in RHS compared to LHS patients during bilateral anti-phase movement is in line with both hemispheric specialization theories on open-loop/close-loop and predictive/impedance movement control [50]. Evidence from both theories argues that the right hemisphere is specialized for sensory-mediated motor control tasks [51–53]. Compared to in-phase, anti-phase movements are usually performed with more errors and variability [46]. This means that increased attentional and executive control, as well as sensory feedback such as error monitoring are needed during anti-phase movements [6], suggesting that the right hemisphere is essential for movement patterns that require higher sensory demands. Besides the theories developed from upper limbs studies, experiments on lower limbs also showed relevant and interesting findings. For instance, RHS compared to LHS patients showed impaired responses to unanticipated perturbations during standing [54], and more asymmetrical gait pattern during walking [55]. These results lead to the view that the right hemisphere is more involved in generating reactive muscular responses in the lower limbs [54]. It is possible that this implication is transferrable to the upper limbs: Patients with impaired right hemisphere maybe disadvantaged in achieving more complex coordination patterns such as anti-phase movements.

### Limitations and outlook

In the current study, we examined the effects of stroke on bilateral coordination on a circle-drawing task required motor control of proximal muscles in the elbow and shoulders. We did not study fine-motor control because many stroke patients are not capable of generating independent finger movements. However, it is important to note that there are neurophysiological and neuroanatomical differences in the control of bilateral proximal limb-coordination vs. bilateral distal-limb coordination [56]. For example, electrical stimulation to forearm muscles on one limb during bilateral hand movement resulted in muscular responses in both limbs, whereas stimulation to one finger during bilateral finger movement resulted in responses only in the stimulated finger. Moreover, recovery outcomes for proximal muscle movements are usually better than that for distal muscles following cortico-spinal tract damage [57]. Therefore there seem to be differences in cortico-muscular coupling and corticospinal contributions between proximal and distal movement control. Furthermore, the human bilateral movement repertoire is very broad, and there exist many other patterns apart from the cyclical rhythmical pattern tested here. Some may be asymmetrical and more complex (such as playing the piano), and some may not be rhythmic (such as opening a bottle) [56]. Another limitation is that we only included participants who were right-handers before stroke. However, handedness could be a factor that influence the results: compared to right-handers, the two hands of the left-handers have a similar level of motor skill performance, and exhibit less effective connectivity between both hemispheres during unilateral movements [58, 59]. Most likely such differences also impact on bilateral coordination, and lateralized control of bimanual coordination might be reduced in these individuals. Therefore, it could be possible that the findings in the current experiment cannot be generalized to patients who were left-handed or mixed-handed before stroke. Another limitation of the current study is the small sample size. Due to practical reasons, we were unable to recruit more patients in our study. However, our statistical approach enabled us to examine effects on the single-trial level and not averaged for each subject as in typical ANOVAs. This allows us to explicitly account for the high variability exhibited by stroke patients to improve the robustness of our results. Nevertheless, with a larger sample, the investigations and discussions can be extended beyond the lesion hemisphere. For example, lesion mapping analysis could be performed to associate more specifically lesioned brain areas with functional impairments [60].

### Implications

Our finding that bilateral anti-phase and in-phase movements are differentially impaired after left and right hemispheric stroke has important implications for neurorehabilitation strategies. Bilateral movement rehabilitation approaches, such as Bilateral Arm Training with Rhythmic Auditory Cueing (BATRAC) and Bilateral Arm training (BBT), have shown inconsistent results in longitudinal studies [61–63]. This could be because these existing trainings usually employed a mixed protocol that included both bilateral in-phase/anti-phase movements. Also, most work in this area has not differentiated trainings for left/right hemispheric stroke patients [61]. Our results suggest that specific bilateral interventions could be tailored to target the different deficits following left/right hemispheric stroke. For example, after right hemispheric stroke, anti-phase movements are more impaired; therefore, patients would benefit from training that focuses on the simultaneous control of non-homologous muscles. The relatively preserved in-phase movements also mean that this movement mode would be a good option for improving timing control, which is important for enhancing the efficiency of daily activities. In contrast, in-phase movements are disturbed after a left hemispheric stroke. Therefore, besides using in-phase movements as a facilitation technique for the contralesional muscles [64], this movement mode should be specifically trained to improve coordination between hands. Once patients could manage these two movement patterns in a clinical setting, additional practical programs should be developed to transfer a task from the practice mode to the real world.

## Conclusions

In conclusion, we found differential impairments in bilateral movements after left and right hemispheric stroke, and proposed distinct neural mechanisms leading to these impairments. These findings provide insights to the development of differential strategies for bilateral coordination training in patients with left and right hemispheric lesions.

### Supplementary Information

**Additional file 1. Material 1**: Arm position matching test. **Material 2**: lesion overlap images. **Material 3**: trajectory variability during unilateral movements. **Material 4**: effects of individual-limb performance on inter-limb synchronization during bilateral anti-phase movements.

## Data Availability

Anonymized data are available upon reasonable request.

## Abbreviations

FM-UE: Fugl-Meyer-Upper Limb score
IHI: Inter-hemispheric inhibition
LHC: Left hemispheric control group
LHS: Left hemispheric stroke group
LMM: Linear mixed-effects models
MAS: Modified Ashworth Scale
MMSE: Mini-Mental Scale Examination
RHC: Right hemispheric control group
RHS: Right hemispheric stroke group
TMS: Transcranial current stimulation
