# Supplementary material for: The impact of lesion side on bilateral upper limb coordination after stroke
Source: J Neuroeng Rehabil. 2023 Dec 13;20:166. doi: 10.1186/s12984-023-01288-4 (PMC10717693; doi:10.1186/s12984-023-01288-4)
Supplement: Supplementary file 1 — Additional file 1. Material 1: Arm position matching test. Material 2: lesion overlap images. Material 3: trajectory variability during unilateral movements. Material 4: effects of individual-limb performance on inter-limb synchronization during bilateral anti-phase movements. [file 12984_2023_1288_MOESM1_ESM.pdf]

Additional file 1

The impact of lesion side on bilateral upper limb coordination after stroke

Pei-Cheng Shih[1,2], Christopher J. Steele[1,3], Dennis Hoepfel[4], Toni Muffel[1,5], Arno Villringer[1,5,6], Bernhard Sehm[1,6,7*]

1. Department of Neurology, Max Planck Institute for Human Cognitive and Brain Sciences, Leipzig, Germany
2. Sony Computer Science Laboratories, Inc., Tokyo, Japan
3. Department of Psychology, Concordia University, Montreal, Quebec, Canada
4. Clinic and Polyclinic for Psychiatry and Psychotherapy, Leipzig, Germany
5. Charité Universitätsmedizin Berlin, Berlin, Germany
6. Department of Cognitive Neurology, University Hospital Leipzig, Leipzig, Germany
7. Department of Neurology, University Hospital Halle (Saale), Halle, Germany

Additional file 1. Material 1: Arm position matching test

We used the Arm Position Matching test provided by the KINARM system to examine participants' proprioceptive ability. Participants sat inside the KINARM, and their visions were blocked. The paradigm is shown in Additional Figure 1. The KINARM moved patient's contralesional arm (i.e., the paretic arm of the patients) to a given position, and after the KINARM reach the target, patient was instructed to move the other arm to the mirror-matched position. After completion, the KINARM moved patient's contralesional arm to the next position, and the procedure repeated for 54 times (9 targets, 6 repetitions for each target). Proprioception error was quantified as the mean absolute position error (unit: cm) between the two hands.

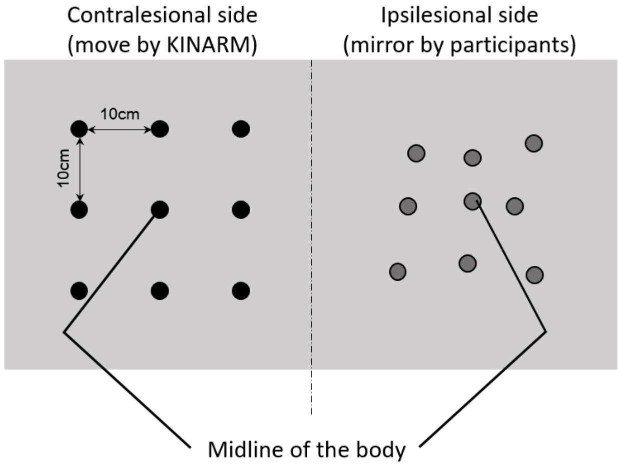

Additional Figure 1. Arm Position Matching test with nine targets.

Additional file 1. Material 2: lesion overlap images

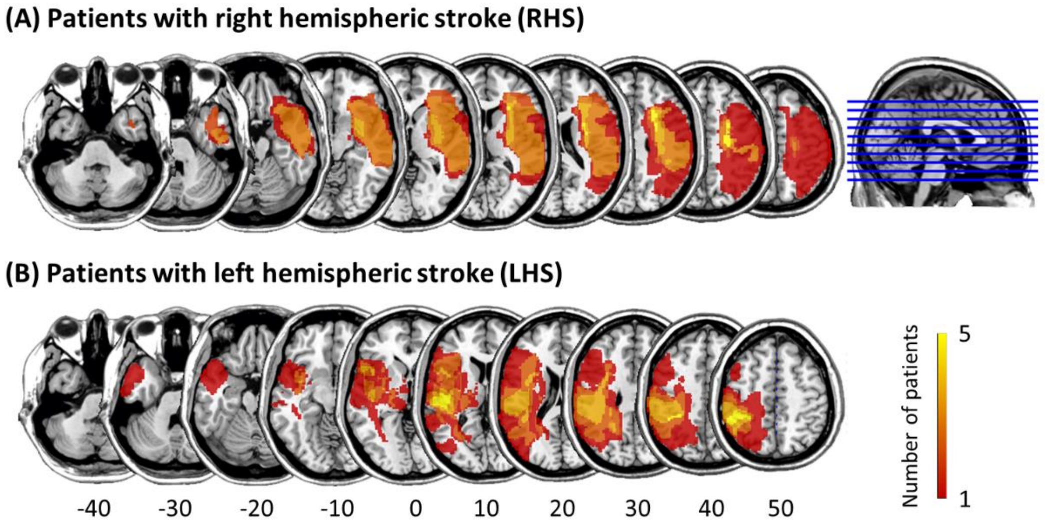

Additional Figure 2. Lesion overlap images for the (A) patients with right hemispheric stroke (RHS) and (B) patients with left hemispheric stroke (LHS) respectively. The number of overlapping lesions is illustrated by the colorbar.

Additional file 1. Material 3: trajectory variability during unilateral movements

As indicated in the manuscript, Figure 2A depicts a representative participant from each group during unilateral condition, and Figure 2B shows the trajectory variability of the contralesional hand on the group level.

We found that stroke patients exhibited higher trajectory variability of the contralesional hand during unilateral movements compared to their control participants in the matched hand as revealed by the significant main effect of Group ($F=23.40$, $p<0.001$), but not Lesioned Hemisphere ($F=1.95$, $p=0.17$) nor Group*Lesioned Hemisphere interaction ($F=0.96$, $p=0.33$). Figure 2C shows the trajectory variability of the ipsilesional hand. The statistical results were similar as in the contralesional performance: there was a significant main effect of Group ($F=8.11$, $p=0.007$), but not Lesioned Hemisphere ($F=3.05$, $p=0.09$) nor Group*Lesioned Hemisphere interaction ($F=0.13$, $p=0.72$). These results indicate that both stroke groups showed impairment in contralesional and ipsilesional hand movements compared to their respective control groups, and the unilateral performance of patients with left and right lesions were not statistically different from each other.

Additional file 1. Material 4: effects of individual-limb performance on inter-limb synchronization during bilateral anti-phase movements

Regression analyses revealed that the performance of the two hands showed similar strength in predicting inter-limb behavior for the control participants and the two stroke groups. For healthy controls, the regression model revealed no significant effect of *Hand* (df=344.62, F=2.82, p=0.09, $\beta_{dominant}$=-0.553, $\beta_{non\text{-}dominant}$=-0.413). For stroke patients, there were no main effects of *Hand* (df=314.53, F=1.96, p=0.16) nor *Lesioned Hemisphere* (df=319.16, F=2.52, p=0.11), and no two-way interaction (df=314.53, F=0.002, p=0.96, $\beta_{RHC,contralesional}$=-0.274, $\beta_{RHC,ipsilesional}$=-0.490, $\beta_{LHC,contralesional}$=-0.350, $\beta_{LHC,ipsilesional}$=-0.249).

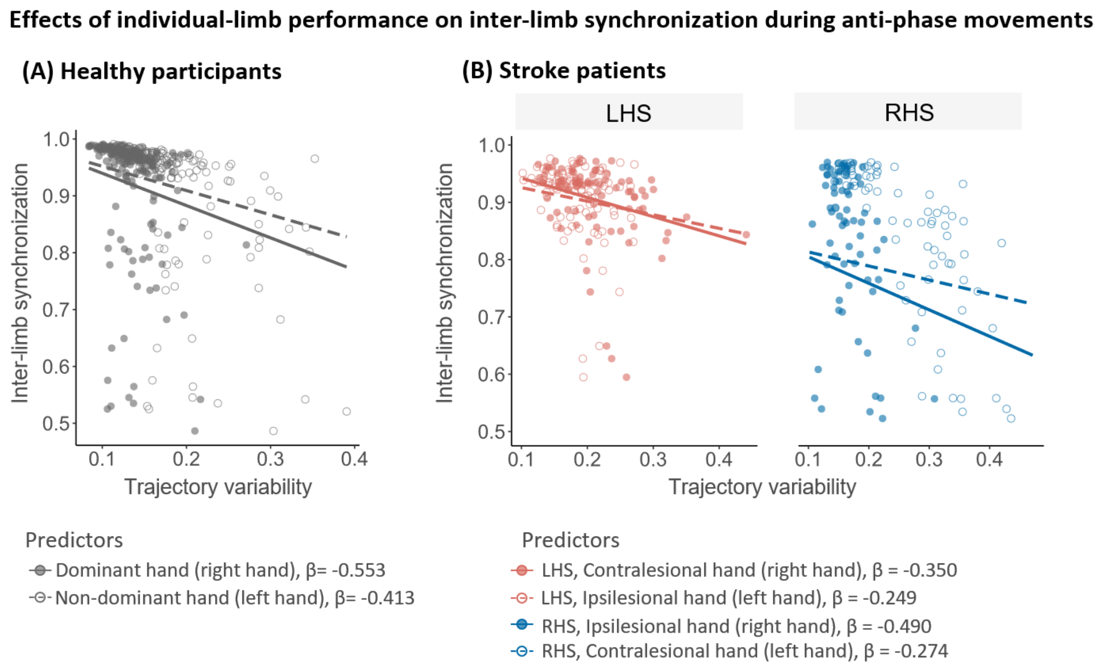

Additional Figure 3. Effects of individual-limb performance on inter-limb synchronization during anti-phase movements. Each dot represents the performance of each trial in each participant.
